# Viewing Romantic and Friendship Interactions Activate Prefrontal Regions in Persons With High Openness Personality Trait

**DOI:** 10.3389/fpsyg.2020.00490

**Published:** 2020-03-24

**Authors:** Atiqah Azhari, Paola Rigo, Pei Yu Tan, Michelle Jin-Yee Neoh, Gianluca Esposito

**Affiliations:** ^1^School of Social Sciences, Nanyang Technological University, Singapore, Singapore; ^2^Department of Developmental Psychology and Socialisation, University of Padua, Padua, Italy; ^3^Department of Psychology and Cognitive Science, University of Trento, Trento, Italy; ^4^Lee Kong Chian School of Medicine, Nanyang Technological University, Singapore, Singapore

**Keywords:** relationships, openness, personality, fNIRS, prefrontal cortex

## Abstract

The personality traits we have and the closeness we experience in our relationships inevitably color the lenses through which we perceive social interactions. As such, the varying perceptions of our social relationships could indicate underlying differences in neural processes that occur in the prefrontal cortex (PFC), a brain region involved in social cognition. However, little is known of how personality traits and relationship closeness with others influence brain responses when viewing social interactions between kin (i.e., siblings) and non-kin (i.e., romantic, friends) partners. In the present study, functional near-infrared spectroscopy (fNIRS) was employed to investigate prefrontal cortical activation patterns in response to three 1-min mute video clips depicting a male–female couple interacting with comparably mild levels of affection while baking, exercising, and eating. The context of the interaction was manipulated by informing participants about the type of relationship each couple in the three video clips was in: (a) romantic partners, (b) friends, or (c) siblings. By changing only the contextual labels of the videos, we revealed distinct PFC responses to relationship type as a function of *openness* trait, closeness with romantic partner, and closeness with siblings. As *openness* score increased, we observed an enhanced activation of the left inferior frontal gyrus (IFG), the left anterior PFC (aPFC), and the right frontal eye fields (FEFs) in response to the video labeled *romantic* and *friendship*, but a reduction in these areas in the *siblings* condition. Similarly, individuals with higher romantic and sibling closeness showed increased activation in the IFG and dorsolateral PFC (dlPFC) in response to *romantic* and *friendship* conditions, but decreased activation in the *siblings* condition. Differences in PFC activations toward romantic, friendship, and sibling relationships reflect underlying variations in the cognitive processing of social interactions, depending on the personality (i.e., *openness*) and experiences (i.e., relationship closeness) of the individual, as well as the relationship type with which the interaction is labeled.

## Introduction

Human affiliations are entrenched in interpersonal love, which has been described as a deep sense of close attachment between two people (Berschied and Peplau, 1983). Depending on whom we share it with, this attachment manifests within varying forms of relationships with kin (i.e., siblings) and non-kin (i.e., friends and partners) individuals. Within low fertility social environments, that is, societies with fertility rates that are lower than the replacement rate of 2.1, where individuals have fewer siblings and cousins, human non-kin relationships are becoming increasingly significant in our lives. The amount of social investment that is required for us to maintain kin and non-kin relationships starkly differs. While the former is perceived to be more stable and granted, the latter requires constant monitoring and personal commitment (Stewart-Williams, 2007; Rotkirch et al., 2014).

Perceptions of social interactions are accompanied by distinct responses in the prefrontal cortex (PFC), a brain area that has been established to occupy an integral role in the interpretation of affective information and in performing higher order socio-cognitive functions (Güroğlua et al., 2008; Cacipoppo et al., 2012; De Boer et al., 2012). Within the medial region of the PFC, the dorsomedial PFC (dmPFC) and ventromedial PFC (vmPFC) networks, in particular, contribute significantly to these processes. For instance, passive viewing of video scenes featuring social interactions between characters was sufficient to significantly elicit dmPFC activity (Wagner et al., 2016). Equally important to affective interpretation is the vmPFC, which has been shown to underscore social judgments of an agent’s capability of possessing a mind (i.e., mind perception). Wiese et al. (2018) found that when participants engaged in a mind perception task that required them to judge the internal mental states of faces which differed in their resemblance to human faces, activity of the vmPFC was found to be significantly associated with mind perception. Aside from the medial networks, the lateral networks of the PFC are also consistently implicated in the regulation of emotions (Ochsner et al., 2012; Tully et al., 2014). For instance, enhanced activation of the ventrolateral PFC (vlPFC) during social exclusion, a form of social stress, is related to lower self-reported ratings of distress (Eisenberger et al., 2003). Given the extensive involvement of prefrontal areas in socio-cognitive processes, we postulate that the PFC is likely to govern differences in perception of social interactions.

Distinct patterns of PFC activation have been found across relationship types as well. For example, in a study that compared the presence of a romantic partner against that of a friend during emotional regulation in response to threatening stimuli, researchers observed greater activation of the vmPFC region in the presence of the romantic partner (Morriss et al., 2018). Their findings suggest that, even in the absence of social interaction, the relationship category of the co-present individual is associated with unique neural responses in the PFC. In another study, Bartels and Zeki (2004) demonstrated that differences in PFC activity was evident between participants who were shown an image of their romantic partner, compared to those who were presented with an image of their child. Heightened activation of the lateral regions of the PFC was observed only for the group that was exposed to images of their child. Similarly, their findings accentuated the pivotal role of the PFC in processing different relationship types. Given the function of the PFC in processing both social interactions and relationship categories, the present study serves as the first to investigate PFC activities when individuals are presented with scenes of social interactions, of comparatively mild affection, labeled with different relationship types.

Personality is defined as one’s characteristic set of thoughts, feelings, and behaviors. There are a number of personality models such as Allport’s trait theory, the Big Five model, and the HEXACO model, that have been proposed (see Cervone and Pervin, 2013; Matz et al., 2016). According to the Big Five model, one of the most dominant and widely used frameworks, personality comprises five core dimensions, namely, *Openness to Experiences (i.e., Openness), Conscientiousness, Extraversion, Agreeableness, and Neuroticism*, which are essential in the interpretation of interpersonal experiences (Hines and Saudino, 2008). In a large representative study, Laakasuo et al. (2017) utilized data from an extensive British Household Panel Survey (*N* = 12,098) to examine the link between an individual’s personality traits and the characteristics of his/her three closest friends. They found that, among the five variables, *openness* was the only trait shown to be correlated to all characteristics of close friends included in the study. For instance, those with higher openness are more likely to have “less traditional friendships,” such as having friends from another country, and possess more friends of the opposite gender. Their findings imply that persons with higher openness trait are likely to establish friendship styles that are exploratory in nature. These results generally signify an association between personality and meaningful differences in the characteristics of one’s close friends. Laakasuo et al. (2017) suggested that the different associations between personality traits and characteristics of close friends could be an indication of varying strategies in the compilation of social networks across individuals. Taken together, these findings suggest the pertinent role of personality traits, *openness* in particular, in influencing non-kin relationships.

Despite the rich body of knowledge in this field, there is a paucity in the investigation of the influence of personality constructs on prefrontal cortical mechanisms of kin and non-kin relationship perception. Compared to more stable kin relationships, non-kin relationships demand greater social investment and attention (Stewart-Williams, 2007; Rotkirch et al., 2014). Little is understood, however, of how differences in social investment moderate distinct perceptions of social interactions between kin and non-kin pairs. Moreover, since the degree of openness was postulated to govern differences in social networking strategies (Laakasuo et al., 2017), there is a possibility that openness would likewise be associated with distinguishing kin from non-kin interactions. To that end, the present study measured the effect of personality variables on prefrontal cortical responses to scenes of kin (i.e., sibling) and non-kin (i.e., friendship and romantic) interactions. Functional near-infrared spectroscopy (fNIRS) offers a sensitive way to record the often nuanced and subtle differences in prefrontal brain responses. Participants were exposed to scenes depicting a male–female pair interacting with comparably mild displays of affection while baking, exercising, and eating. While the order of presentation of video stimuli remained the same, the label attached to the video, either *romantic partners*, *siblings*, or *friendship*, differed across participants. Although the primary focus of the study is on the distinction between kin and non-kin relationships, the non-kin category was further subdivided into *romantic partners* or *friendship* to account for the comparatively greater physical intimacy that is typically expected of the former relationship type. We embarked on this experiment with three hypotheses in mind. First, we anticipated a distinction in medial and lateral PFC activity in response to kin (i.e., sibling) and non-kin (i.e., friendship and romantic) relationships as a function of *openness*. Given that our participants are young adults in a contemporary low fertility society who are likely to invest in the maintenance of previously established friendships while pursuing romantic relationships (Arnett, 2004), and that *openness* is the strongest predictor of traits in friendships, it is likely that PFC activation patterns in response to kin and non-kin interactions differ depending on one’s level of *openness*. Second, since the intensity of affect among siblings follows a linearly decreasing trend into adulthood, whereas that of friends shows an opposite positive trend (Bradac, 1983), we expect that the activities of medial and lateral PFC would depict an inverse relationship between kin and non-kin interactions as a function of relationship closeness. It would, however, be naïve to assume that all kin and non-kin relationships conform to this common trend. While most young adults may veer toward the company of friends and the pursuit of romantic partners, some may nonetheless find comfort with their existing sibling relationships. To account for such individual differences, a measure of relationship closeness across each of the three relationship types would also be obtained. Thus, our third hypothesis is that PFC responses to kin and non-kin interactions may differ as a function one’s closeness level in each relationship type.

## Materials and Methods

### Participants

A total of 44 heterosexual women (*M* = 21.2 years, *SD* = 1.66) and 25 men (*M* = 21.4 years, *SD* = 1.61) were recruited either as paid participants or psychology undergraduates compensated with course credits. The study was approved by the ethics committee and informed consent was obtained from all participants prior to the study. Information regarding participants’ demographic data can be found in Table 1. A preliminary data analysis was conducted to determine whether there were significant group differences between participants in terms of the types of relationships they had. Welch’s *t*-test analyses were conducted on the openness scores of the following groups of participants: (i) with and without siblings of the opposite gender (*t* = 0.311, *df* = 60, *p* = 0.757); (ii) with and without at least one past romantic partner (*t* = −1.912, *df* = 67, *p* = 0.060); (iii) who are currently in a romantic relationship compared to those who are not (*t* = 1.091, *df* = 45, *p* = 0.281), and (iv) across male and female sex (*t* = −0.002, *df* = 67, *p* = 0.998). Median split followed by *t*-tests were also conducted on the age of participants (median = 21, *t* = −0.147, *df* = 67, *p* = 0.883) and number of siblings that participants have (median = 1, *t* = −0.267, *df* = 67, *p* = 0.790). As no significant group differences were found, all individuals in the sample were treated as a group accordingly.

**TABLE 1:** Participants’ demographic information.

Demographics	Frequency
**Gender**	
Male	25
Female	44
**Age (years)**	
18	1
19	15
20	7
21	14
22	16
23	10
24	5
25	1
**Number of siblings**	
0	7
1	34
2	24
3	3
4	1
**Siblings of the opposite gender**	
Yes	38
No	24
**At least one past romantic relationship**	
Yes	47
No	22
**Currently in romantic relationships**	
Yes	26
No	21

#### Questionnaire

##### Personality Questionnaire

Participants were required to complete a personality questionnaire prior to attending the experimental session. The Big Five questionnaire is a 50-item self-report questionnaire on a five-point Likert scale, which requires the participant to report how accurate a sentence is (from 1 = very inaccurate to 5 = very accurate) in describing them (John et al., 1991). The Big Five questionnaire consists of five personality dimensions—*Openness* to experience, *Conscientiousness, Extraversion, Agreeableness*, and *Neuroticism* (Digman, 1990). When administered in college settings, internal consistency measures found this questionnaire to be reliable, with Cronbach’s α of over 0.70 for each trait (Ward, 2017). In our sample, the Cronbach’s α for *Openness* is 0.817.

##### Personal Relationship Closeness Questionnaire

Given that numerous external variables shaped social relationships, we recognized that across individuals, the perception and experience of a particular relationship would differ regardless of whether the relationship was kin or non-kin in nature. Hence, personal relationship closeness (Personal-RC) was administered to account for individual differences in social relationships as a function of how close they perceive these relationships to be. The Personal-RC questionnaire is adapted from the Relationship Closeness Inventory (RCI) (Berscheid et al., 1989) with regard to the relationships of participants with their romantic partners, friends, and siblings. For the friendship subscale, participants were asked to respond regarding their “closest friend” in the questionnaire as follows: “This section consists of questions regarding you and your friendships. Think of your closest friend while answering the following questions.” This inventory consists of one six-point Likert scale item “What is/was/will be the average amount of time you spend with each other per week (in hours)” as well as two five-point Likert scale items “How much influence do you think this person has in your everyday decision-making?” and “How much influence do you think this person has in your important life events?.” An open-ended item regarding the duration of acquaintance was also included “How long have you known this person for (in years)?.” In our sample, the Cronbach’s α for closeness with romantic partner, friends, and siblings are 0.857, 0.772, and 0.865, respectively.

### Experimental Design

**FIGURE 1:**
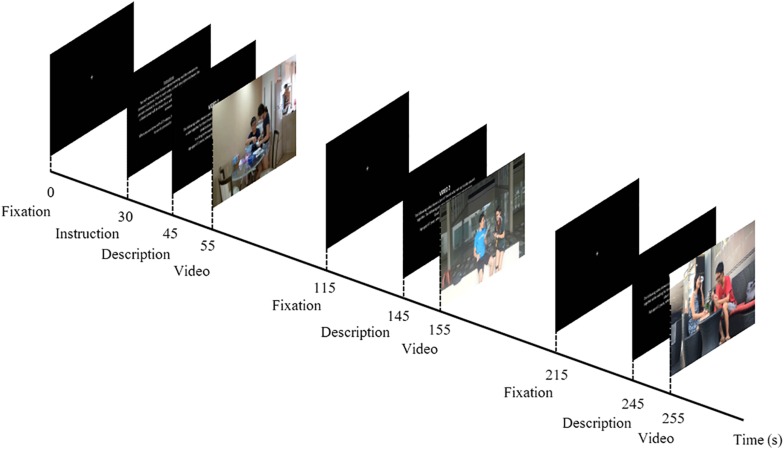
Schematic diagram of experimental design. At the start of the experiment, a fixation cross was displayed against a black screen for 30 s, and this was followed by the instructions for the task, which lasted for 15 s. A description of the first video was screened for 10 s before the onset of the first video stimulus. Each video clip was screened for a duration of 60 s, which was subsequently followed by a recovery period (i.e., fixation cross) of 30 s before the description of the next video was depicted. A total of three video clips were shown, depicting a male–female pair interacting in the following activities: (i) baking, (ii) exercising, and (iii) eating. While the order of activities that the male–female pair engaged in was fixed (i ii iii), the description of the type of relationship corresponding to the video, (a) Romantic, (b) Friendship, and (c) Siblings, was counterbalanced across participants: Romantic–Friends–Siblings (a b c), Friends–Siblings–Romantic (b c a), and Siblings–Romantic–Friend (c a b).

Participants were seated alone in a dimly lit room and presented with a series of three videos on a 15-inch screen laptop PC, along with a randomized description of the actors’ relationship in each video. At the start of the experiment, a fixation cross against a blank screen was shown to the participant for 30 s, before a 15-s instruction page was displayed. A short description of the relationship between the male–female pair, (a) Romantic partners, (b) Friendship, and (c) Siblings, and the activity within the video, (i) baking, (ii) exercising, and (iii) eating, were shown on the screen for 10 s before the onset of the video stimuli. An inter-stimulus interval (ISI) of 30 s preceded the 10-s description of the subsequent video. Likewise, a recovery period of 30 s, followed by a 10-s description, occurred before the onset of the final video (Figure 1).

The mute videos were shown in the same order (i ii iii) to all participants but the relational context in which the interaction occurred was manipulated by informing participants of the nature of the relationship. Prior to the experimental session, participants were randomly assigned into three groups, where they were told that the relationship corresponding to the videos were as follows: a b c; b c a; and c a b. This experimental design fixed the order of activity of the videos across all participants (i ii iii), while changing only the description of the relationship type matched to the videos.

#### Video Stimuli

A digital video recording of the three stimuli was performed using an OPPO video camera. Three different pairs of opposite-gendered actors, of Chinese ethnicity, were recruited to engage in similar levels of mild displays of affection which was filmed in three separate videos. All videos were filmed from the same angle and distance, showing only the two actors and no other persons. In the video, actors interacted with each other in the following social contexts: (i) while baking together, (ii) while exercising together, and (iii) while eating together. The videos were edited to control for visual parameters (i.e., brightness, hue) and all sounds were removed. The duration of each clip was cut to 60 s.

During the experiment, the relational context of each video was manipulated by changing the description of the relationship of the actors given to the participants. We conducted a pilot test of the videos to ensure that this manipulation was valid. Six different videos were filmed and a focus group discussion was held with the participants of the pilot test (*n* = 10) to decide on the videos to be used in the study. We asked participants in the pilot test regarding the (i) plausibility of these activities occurring between individuals in the three types of relationships tested (romantic partners, friends, and siblings) and (ii) extent to which they believed that the actors in each video could be thought of as being either romantic partners, friends, or siblings. The final videos used as the experimental stimuli were those agreed by participants during the focus group discussion to have met the following criteria: (i) activities could occur between individuals in the three types of relationships and (ii) actors could be believed to be either romantic partners, friends, or siblings.

### Functional Near-Infrared Spectroscopy (fNIRS) Data Acquisition

As participants viewed the videos, data were recorded using a functional NIRS imaging system (NIRSport, NIRx Medical Technologies LLC, Glen Head, NY, United States) with eight LED-sources and seven detectors, corresponding to a 20-channel montage of the PFC. Dual wavelengths of 760 and 850 nm were used to measure hemodynamic changes in oxygenated (HbO) and deoxygenated (HbR) blood. The signal was recorded at a sample rate of 7.81 Hz on NIRStar Software 14.0.^1^ NIRS allows for the monitoring of localized changes in blood oxygenation which serves as a proxy of brain activation. Signal quality was adjusted and calibrated on NIRStar prior to the start of the experiment. The dataverse for this study has been published at: https://doi.org/10.21979/N9/TSVWRR.

### NIRS Pre-Processing and Analyses

NIRS data were pre-processed using NIRSLab ver. 2016.^1^ Discontinuities were removed, and spikes were identified via visual inspection and replaced with signals nearest to the spike artifacts. Channels with significant noise (gain > 8 and CV > 7.5) were excluded from further pre-processing. A bandpass filter of 0.1–0.2 Hz was applied to eliminate any physiological slow signal and baseline shift variations. Following that, hemodynamic states were measured using a modified Beer–Lambert Law with differential pathlength factor (DPF) of 7.25 and 6.38 for 760 and 850 nm wavelengths, respectively.

Analyses of the pre-processed NIRS data were conducted at two levels: within-subject analysis (first-level) and group-level analysis (second-level). At the first level of analysis, beta-coefficients for each of the relationship conditions were extracted from the GLM of each individual participant. The GLM was based on a hemodynamic response function (HRF) setting and followed a Gaussian full-width at half-maximum (FWHM) 4 model. Discrete cosine transform (DCT) function with a high-pass period cut-off of 128 s was applied to the matrix before the beta-coefficients were obtained.

At the second level of analysis, beta-coefficients from each participant were combined into group-level GLMs. To test the first hypothesis, that *openness* moderates PFC activation differently in response to *romantic partners*, *friends*, and *sibling* conditions, five GLMs were conducted on each channel to investigate the significance of each personality dimension. The dependent variable in each model was the beta-coefficient values, while the independent variable was the relationship condition and participants’ personality score (i.e., beta-coefficient ∼ relationship condition ^∗^ *openness*). GLM analyses were conducted on each of the 20 channels. Since the results were corrected for a large number of multiple comparisons across channels, each personality dimension was tested in a separate GLM to reduce the degrees of freedom in each model. To test the second hypothesis, that an inverse trend of prefrontal responses would be observed for kin and non-kin relationships as a function of *openness*, Pearson’s product–moment correlation test would be conducted for channels which emerged to be significant from the GLM analyses.

To test the third hypotheses, that relationship closeness moderates PFC activation differently in response to *romantic partners*, *friends*, and *sibling* conditions, three GLMs were conducted for each channel, where the independent variables were participants’ *romantic closeness* (i.e., beta-coefficient ∼ relationship condition ^∗^ *romantic closeness*), *friendship closeness* (i.e., beta-coefficient ∼ relationship condition ^∗^ *friendship closeness*), and *siblings closeness* (i.e., beta-coefficient ∼ relationship condition ^∗^ *siblings closeness*). First, false discovery rate (FDR) correction was applied across 20 channels (Benjamini and Hochberg, 1995) to account for multiple comparisons so as to obtain a corrected *p*-value for each channel. Next, each of these corrected *p*-values were compared against the new critical *p*-value for each channel (*p* = 0.0167) which was Bonferroni corrected. Only FDR corrected *p*-values that survived Bonferroni correction would be reported as significant. Pearson’s product–moment correlation test would also be conducted on significant channels to determine the direction of effect of relationship closeness on PFC activity.

## Results

### Relationship Type and *Openness*

A generalized linear model (GLM) analysis was conducted on the HbO beta-coefficients (*relationship type* as within-participant factor and *openness* as covariate). Significant *relationship type* and *openness* interaction, which survived multiple comparisons correction, was obtained in the left inferior frontal gyrus [IFG, BA45L—Channel 3, *F*_(__2_,_189__)_ = 3.117, corrected *p* = 0.0138, η_*p*_^2^ = 0.032], the left anterior PFC [aPFC, BA10L—Channel 6, *F*_(__2_,_192__)_ = 6.543, corrected *p* = 0.0138, η_*p*_^2^ = 0.064], and the right frontal eye field [FEF, BA08R—Channel 10, *F*_(__2_,_162__)_ = 8.943, corrected *p* = 0.00414, η_*p*_^2^ = 0.099). No main effects of *relationship type* and *openness* emerged.

#### *Relationship Type* and *Openness* Interaction

**FIGURE 2:**
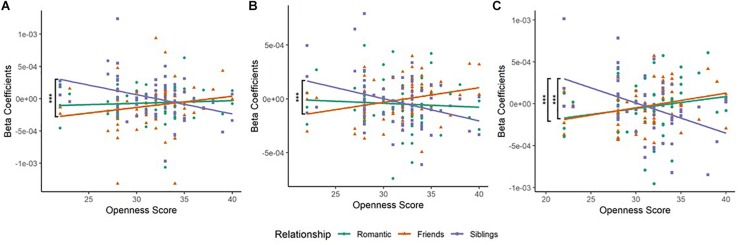
**(A)** Scatterplot of Relationship type and Openness score in the left inferior frontal gyrus (BA45L—Channel 3). Pearson’s product–moment correlations were conducted for each relationship type with openness score: *Romantic*–Openness (RO), *Friendship*–Openness (FO), and *Sibling*–Openness (SO). The difference in correlation coefficients between FO and SO was significant (*Z* = 3.17, *p* = 0.0015). This was observed in the left inferior frontal cortex (IFC). **(B)** Scatterplot of Relationship type and Openness score in left anterior Prefrontal Cortex (BA10L—Channel 6). Pearson’s product–moment correlations were conducted for each relationship type with openness score. The difference in correlation coefficients between FO and SO was significant (*Z* = 3.6, *p* = 0.0003). This was observed in the left anterior prefrontal cortex. **(C)** Scatterplot of Relationship type and Openness score in the right frontal eye field (BA08R—Channel 10). Pearson’s product–moment correlations were conducted for each relationship type with openness score. The difference in correlation coefficients between FO and SO (*Z* = 3.72, *p* = 0.0002), and RO and SO (*Z* = 3.14, *p* = 0.0017) were significant. This was observed in the right frontal eye fields. *p < 0.05, **p < 0.01, ***p < 0.001.

(a) In the left IFG (BA45L—Channel 3), Pearson’s product–moment correlation revealed a negative correlation between *siblings condition* and *openness* (SO; *r* = −0.364, *t* = −3.097, *df* = 63, *p* = 0.003, power = 0.85). The correlations between *romantic condition* and *openness* (RO; *r* = 0.067, *t* = 0.531, *df* = 63, *p* = 0.597, power = 0.082), and *friendship condition* and *openness* (FO; *r* = 0.191, *t* = 1.54, *df* = 63, *p* = 0.128, power = 0.33) were not found to be significant. To evaluate the significance of the difference between two correlation coefficients, a Fisher r-to-z transformation was applied. From this analytical step, only the correlation coefficients between FO and SO was found to be significant (*Z* = 3.17, *p* = 0.002; Table 2A and Figure 2A). No significant difference was observed between the correlation coefficients of RO and SO, as well as RO and FO.

**TABLE 2:** Table depicting significant channels, associated brain areas, *r*-values of correlations, and *Z-* and *p*-values of Fisher’s test of difference between two correlation coefficients. **(A)** Correlation between Relationship condition (*romantic, friendship, siblings*) and *Openness*, **(B)** Correlation between Relationship condition (*romantic, friendship, siblings*) and *Romantic Closeness*, and **(C)** Correlation between Relationship condition (*romantic, friendship, siblings*) and *Siblings Closeness*.

**(A)**										
Brain region		*r*			*Z*			*p*		
Channel	Corresponding area	*Romantic*-Openness (RO)	*Friendship*-Openness (FO)	*Siblings*-Openness (SO)	RO-FO	RO-SO	FO-SO	RO-FO	RO-SO	FO-SO
CH3	Left inferior frontal gyrus (IFG)	0.067	0.191	−0.366**	–0.7	2.47	3.17	0.4839	0.0135	0.0015**
CH6	Left anterior prefrontal cortex (aPFC)	−0.065	0.281*	−0.344**	–1.97	1.63	3.6	0.0488	0.1031	0.0003***
CH10	Right frontal eye field (FEF)	0.173	0.282*	−0.414**	–0.59	3.14	3.72	0.5552	0.0017**	0.0002***

**(B)**										

**Brain region**		***r***			***Z***			***p***		
**Channel**	**Corresponding area**	***Romantic*-Romantic Closeness (RR)**	***Friendship*-Romantic Closeness (FR)**	***Siblings*-Romantic Closeness (SR)**	**RR-FR**	**RR-SR**	**FR-SR**	**RR-FR**	**RR-SR**	**FR-SR**

CH3	Left inferior frontal gyrus (IFG)	0.163	0.120	−0.445***	0.24	3.55	3.31	0.8103	0.0004***	0.0009***
CH15	Right lateral dorsolateral prefrontal cortex (DLPFC)	0.117	0.243	−0.535***	–0.53	2.9	3.43	0.5961	0.0037**	0.0006***

**(C)**										

**Brain region**		***r***			***Z***			***p***		
**Channel**	**Corresponding area**	***Romantic*-Sibling Closeness (RS)**	***Friendship*-Sibling Closeness (FS)**	***Siblings*-Sibling Closeness (SS)**	**RS-FS**	**RS-SS**	**FS-SS**	**RS-FS**	**RS-SS**	**FS-SS**

CH1	Left middle frontal gyrus (MFG)	0.164	0.129	−0.471***	0.19	3.61	3.42	0.8493	0.0003***	0.0006***

*p < 0.05, **p < 0.01, ***p < 0.001.

(b) In the left aPFC (BA10L—Channel 6), Pearson’s product–moment correlation revealed a negative correlation between *siblings condition* and *openness* (SO; *r* = −0.344, *t* = −2.93, *df* = 64, *p* = 0.005, power = 0.811), and a positive correlation between *friendship condition* and *openness* (FO; *r* = 0.281, *t* = 2.34, *df* = 64, *p* = 0.022, power = 0.628). Fisher r-to-z transformation was applied, producing a significant difference between the correlation coefficients of FO and SO (*Z* = 3.6, *p* = 0.0003; Table 2A and Figure 2B). The correlation between *romantic condition* and *openness* (RO; *r* = −0.065, *t* = −0.52, *df* = 64, *p* = 0.605, power = 0.081) was not found to be significant. No significant difference was observed between the correlation coefficients of RO and SO, as well as RO and FO.

(c) In the right FEF (BA08R—Channel 10), Pearson’s product–moment correlation revealed a positive correlation between *friendship condition* and *openness* (FO; *r* = 0.282, *t* = 2.163, *df* = 54, *p* = 0.035, power = 0.559), and a negative correlation between *siblings condition* and *openness* (SO; *r* = −0.414, *t* = −3.342, *df* = 54, *p* = 0.002, power = 0.893). Fisher r-to-z transformation was applied, producing a significant difference between the correlation coefficients of FO and SO (*Z* = 3.72, *p* = 0.0002), as well as RO and SO (*Z* = 3.14, *p* = 0.002; Table 2A and Figure 2C). The correlation between *romantic condition* and *openness* (RO; *r* = 0.173, *t* = 1.29, *df* = 54, *p* = 0.201, power = 0.247) was not significant. No significant difference was observed between the correlation coefficients of RO and FO.

No main effect of *relationship type*, and no main effect of the other four personality variables (i.e., *conscientiousness, extraversion, agreeableness, neuroticism*), or their two-way interaction was found.

### Relationship Type and *Romantic Closeness*

Similarly, a GLM analysis was conducted on the HbO beta–coefficients (*relationship type* as within-participant factor and *romantic closeness* as covariate). Significant *relationship type* and *romantic closeness* interaction, which survived correction, was obtained in the left IFG [BA45L—Channel 3, *F*_(__2_,_189__)_ = 8.099, corrected *p* = 0.0082, η_*p*_^2^ = 0.079] and the right lateral dorsolateral PFC [dlPFC, BA09R—Channel 15, *F*_(__2_,_105__)_ = 7.610, corrected *p* = 0.0082, η_*p*_^2^ = 0.127]. No main effects of *relationship type* and *romantic closeness* emerged.

#### *Relationship Type* and *Romantic Closeness* Interaction

**FIGURE 3:**
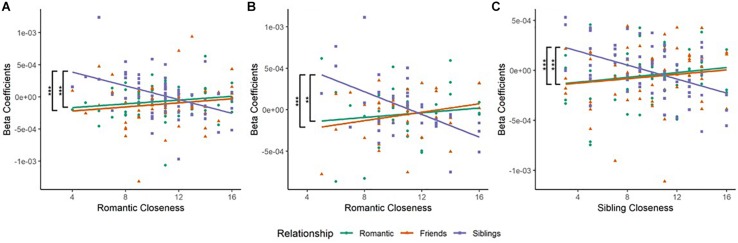
**(A)** Scatterplot of Relationship type and Romantic Closeness score in the left Inferior Frontal Gyrus (BA45L—Channel 3). Pearson’s product–moment correlations were conducted for each relationship type with openness score: *Romantic*–Romantic Closeness (RR), *Friendship*–Romantic Closeness (FR), and *Sibling*–Romantic Closeness (SR). The difference in correlation coefficients between RR and SR was significant (*Z* = 3.55, *p* = 0.0004). The difference in correlation coefficients between FR and SR was also significant (*Z* = 3.31, *p* = 0.0009). These observations were mapped to the left inferior frontal gyrus (IFG). **(B)** Scatterplot of Relationship type and Romantic Closeness score in the right Lateral Dorsolateral PFC (BA09R—Channel 15). Pearson’s product–moment correlations were conducted for each relationship type with openness score. The difference in correlation coefficients between RR and SR was significant (*Z* = 2.9, *p* = 0.0037). The difference in correlation coefficients between FR and SR was also significant (*Z* = 3.43, *p* = 0.0006). These observations corresponded to the right lateral dorsolateral prefrontal cortex (DLPFC). **(C)** Scatterplot of Relationship type and Sibling Closeness score in the left middle frontal gyrus (MFG, BA46L—Channel 1). Pearson’s product–moment correlations were conducted for each relationship type with openness score: *Romantic*–Sibling Closeness (RS), *Friendship*–Sibling Closeness (FS), and *Sibling*–Sibling Closeness (SS). The difference in correlation coefficients between RS and SS was significant (*Z* = 3.61, *p* = 0.0003). The difference in correlation coefficients between FS and SS was also significant (*Z* = 3.42, *p* = 0.0006). These results were mapped to the left MFG. *p < 0.05, **p < 0.01, ***p < 0.001.

(a) In the left IFG (BA45L—Channel 3), Pearson’s product–moment correlation revealed a negative correlation between *siblings condition* and *romantic closeness* (SR; *r* = −0.445, *t* = −3.941, *df* = 63, *p* = 0.0002, power = 0.964). The correlations between *romantic condition* and *romantic closeness* (RR; *r* = 0.163, *t* = 1.315, *df* = 63, *p* = 0.193, power = 0.254), and between *friendship condition* and *romantic closeness* (FR; *r* = 0.12, *t* = 0.959, *df* = 63, *p* = 0.341, power = 0.157) were not significant. Fisher r-to-z transformation revealed significant differences between the correlation coefficients of RR and SR (*Z* = 3.55, *p* = 0.0004) as well as FR and SR (*Z* = 3.31, *p* = 0.001; Table 2B and Figure 3A). No significant difference was observed between the correlation coefficients of RR and FR.

(b) In the right lateral dlPFC (BA09R—Channel 15), Pearson’s product–moment correlation revealed a negative correlation between *siblings condition* and *romantic closeness* (SR; *r* = −0.534, *t* = −3.746, *df* = 35, *p* = 0.001, power = 0.935). Correlations between *romantic condition* and *romantic closeness* (RR; *r* = 0.118, *t* = 0.7, *df* = 35, *p* = 0.489, power = 0.105], and between *friendship condition* and *romantic closeness* (FR; *r* = 0.244, *t* = 1.485, *df* = 35, *p* = 0.147, power = 0.303) were not significant. Applying the Fisher r-to-z transformation, we found a significant difference between the coefficients of RR and SR (*Z* = 2.9, *p* = 0.004) as well as FR and SR (*Z* = 3.43, *p* = 0.001; Table 2B and Figure 3B).

### Relationship Type and *Sibling Closeness*

A GLM analysis was conducted on the HbO beta–coefficients (*relationship type* as within-participant factor and *sibling closeness* as covariate). Significant *relationship type* and *sibling closeness* interaction was obtained in the left middle frontal gyrus [MFG, BA46L—Channel 1, *F*_(__2_,_177__)_ = 7.626, corrected *p* = 0.01332, η_*p*_^2^ = 0.079]. No main effects of *relationship type* and *sibling closeness* emerged.

#### *Relationship Type* and *Sibling Closeness* Interaction

(a) In the left MFG (BA46L—Channel 1), Pearson’s product–moment correlation revealed a negative correlation between *siblings condition* and *romantic closeness* (SR; *r* = −0.471, *t* = −4.097, *df* = 59, *p* = 0.0001, power = 0.973). Correlations between *romantic condition* and *sibling closeness* (RS; *r* = 0.164, *t* = 1.276, *df* = 59, *p* = 0.207, power = 0.242) and between *friendship condition* and *romantic closeness* (FS; *r* = 0.123, *t* = 0.998, *df* = 59, *p* = 0.322, power = 0.166) were not significant. Applying Fisher r-to-z transformation, we found a significant difference between the coefficients of RS and SS (*Z* = 3.61, *p* = 0.0003) as well as FS and SS (*Z* = 3.42, *p* = 0.0006; Table 2C and Figure 3C). No significant difference was observed between the correlation coefficients of RS and FS.

### Relationship Type and *Friendship Closeness*

A GLM analysis was conducted on the HbO beta–coefficients (*relationship type* as within-participant factor and *friendship closeness* as covariate). No significant main effect of *relationship type* or *friendship closeness*, or their two-way interaction was found.

## Discussion

The principal aim of this study was to investigate the difference in PFC activation when participants viewed social interactions between male–female kin and non-kin pairs, as a function of personality traits and relationship closeness. The first hypothesis, that variation in level of *openness* will govern distinct medial and lateral PFC activities in response to non-kin (i.e., friendship and romantic) and kin (i.e., sibling) interactions, was fulfilled. The second hypothesis was also satisfied as we found an inverse pattern of cerebral activation that emerged in the left IFG (BA45), left aPFC (BA10), and right FEF (BA8) when viewing *friendship* and *romantic* interactions (i.e., non-kin) compared to *sibling* (i.e., kin) interactions, depending on the *openness* level of the participant. Individuals with higher *openness* trait showed significantly greater activation toward *romantic* than *siblings* condition in the IFG and aPFC. Additionally, those with a higher level of *openness* also exhibited significantly greater activation toward *romantic* and *friendship* conditions compared to the *siblings* condition in the FEF. While the IFG and aPFC fall within the dmPFC and vmPFC networks, respectively, the FEF is located within the ventrolateral network (vlPFC). No other personality trait was found to be significantly related to brain responses when viewing scenes of different relationship categories.

The third hypothesis, that relationship closeness will lead to distinct medial and lateral PFC activation patterns in response to non-kin and kin interactions, was also fulfilled. Similarly, an inverse pattern emerged in response to non-kin (i.e., romantic, friendly) and kin (i.e., sibling) interactions as a function of *romantic closeness* and *sibling closeness*. We found that individuals with higher *romantic closeness* showed greater activation in the left IFG, part of the dorsomedial network, and the right dlPFC, toward *romantic* and *friendship* condition compared to the *siblings* condition. Moreover, those with higher *sibling closeness* exhibited greater activation in the left MFG, part of the dlPFC network, in response to *romantic* and *friendship* conditions compared to the *siblings* condition. No significant effect of *friendship closeness* was found.

### *Openness* and the Social Brain

Among all other personality dimensions, *openness* most potently governs the development of friendships, where the ideal friend is described to have the same level of *openness* as the individual (Cheng et al., 1995). As individuals enter adolescence and young adulthood, mild displays of affection among siblings also occur less frequently (Bradac, 1983; Pulakos, 1989). This pattern of socialization is particularly observed in contemporary low fertility societies where emerging adulthood connotes that one consistently interacts with non-kin relations such as friends, rather than kin relations such as siblings, on a daily basis. These findings were later corroborated in a recent study by Laakasuo et al. (2017) who revealed that *openness*, rather than other personality traits, predicted all characteristics of a young adult’s closest friends.

Given the pertinent role of *openness* in the development of non-kin relationships, our finding that the level of *openness* is associated with an inverse activation pattern of the IFG and aPFC toward friendly compared to sibling interactions offers a remarkable insight into the mechanisms by which *openness* influences relationship perception. The IFG and aPFC are both located within the larger dmPFC and vmPFC networks, which are known to be recruited for interpretation of social interactions and higher order social cognition, such as making perceptual judgments regarding the mental states of others (Iacoboni et al., 2005; Cleeremans et al., 2007; Schulte-Rüther et al., 2007). Greater activation of the medial PFC in individuals with higher levels of *openness* suggests that they recruited more cognitive resources for affective interpretation of mental states of actors when they were labeled as friends compared to when they were labeled as siblings. It may be possible that persons higher on *openness*, who are prototypically used to having less “traditional” friendships and possess a variety of friends, including more friends from the opposite gender (Selfhout et al., 2010; Laakasuo et al., 2017), engaged in more flexible perceptual assessments when viewing non-kin interactions which was reflected in the brain as greater activation of the medial PFC.

Compared to the medial regions of the PFC, which only distinguished between *friendship* and *siblings* conditions, the ventrolateral region of the PFC, in which the FEF is located, showed an inverse association between both categories of non-kin relationships (i.e., *romantic, friendly*) and kin relationship (i.e., *siblings*). The vlPFC is primarily involved in emotional regulatory processes (e.g., Ochsner et al., 2012). Thus, the distinct pattern of activation in response to non-kin and kin relations that emerged here potentially signals differences in regulatory mechanisms of individuals with higher compared to lower levels of *openness*. Kin and non-kin relations differ fundamentally in the extent of psychological maintenance required of them. While kin interactions are more instrumental and robust, non-kin interactions typically provide greater emotional support despite degrading quickly in the absence of constant social investment (Park and Ackerman, 2011; Roberts and Dunbar, 2011). Compared to their counterparts who scored lower on *openness*, individuals with higher *openness* tend to establish warmer relationships with their siblings (Walęcka-Matyja, 2018). Having safeguarded their “default” kin relationships, individuals with higher *openness* might afford to invest in “chosen” non-kin relationships. Due to their stable kin relationships that demand less social maintenance, more *open* individuals could have required greater emotional regulation only when viewing affectionate interactions between non-kin dyads, whereas less regulatory resources could have been recruited in response to the *sibling* condition.

### *Relationship Closeness* and the Social Brain

With a higher level of *romantic closeness*, greater activation in the *romantic* and *friendship* conditions was observed in the left IFG, situated within the dmPFC, and the right dlPFC. These dorsal regions are implicated in the processing of contextualized social information (Carr et al., 2003; Shamay-Tsoory et al., 2009; Keysers et al., 2010; Schurz et al., 2014) and higher order social cognition, including social perspective-taking and inferring the intentions of others (Miller and Cummings, 2007). In the *romantic* condition, it is likely that greater closeness with romantic partners led participants to enhance the recruitment of these regions for processing of social information in a romantic context. Interestingly, this elevated pattern of activity emerged in the friendship condition as well. Drawing upon kin theories, one postulation is that individuals who are in love are likely to attend to stimuli that encapsulate potential threats in mating, such as the affection shown between non-kin friends of opposite genders. Alternatively, greater activation in the *friendship* condition might simply indicate that processing of social interaction in the context of friendship may be influenced by one’s romantic experiences.

An enhanced activation of the dlPFC in both *romantic* and *friendship* conditions might also allude to the possibility that more cognitive resources were required to distinguish between the two complex overlapping relationship types (Backman and Secord, 1959; Sprecher, 1998). Intriguingly, an inverse association was observed in the *siblings* condition, in which a higher level of *romantic closeness* was associated with reduced activation in the dmPFC and dlPFC. This suggests that *romantic closeness* configures an important basis upon which social perceptions of friendship and romantic interactions are formed, both of which are distinct from sibling interactions. Lastly, compared to the *siblings* condition, an unambiguous pattern of similarity between friendship and romantic conditions emerged as a function of *siblings closeness* too. These consistent findings lend support to the notion that kin and non-kin interactions are processed differently in the prefrontal region of the brain.

### Future Directions

Although personality represents the main focus of this study, experiential factors in each of these three relationships were investigated by analyzing *relationship closeness*. Comparing *openness* and *relationship closeness*, both analyses revealed a similar negative trend in the *siblings condition*, where higher closeness and openness scores were associated with reduced activation. Moreover, the generally positive correlation between *closeness* and *friendship condition*, and *closeness* and *romantic condition*, paralleled the trend seen as a function of *openness*. This observation brings to bear the question on how personality and past experiences dually operate to elicit a similar influence on the neural events that underscore differential perceptions of relationships. One possible postulation is that there exists an intrinsic link between openness and relationship closeness. Indeed, persons who are more open tend to experience less discord with others, which aids in attaining greater intimacy and closeness in their relationships (Berry et al., 2000). Further studies are required to fully explicate the dynamic effects of personality and experiences on the perceptions of relational interactions.

### Limitations and Conclusion

We have revealed the rich influence of the personality trait *openness* in influencing PFC responses to stimuli of different relationship conditions. However, several limitations of this study should be addressed. First, given the methodical limitation of the NIRS device, this study only focused on the prefrontal areas of the brain and marked differences may indeed exist in other cortical or subcortical areas of the brain. Second, subjective behavioral responses of participant ratings on interpersonal parameters of the couples in the videos, such as level of warmth, likeability, affection, and reciprocity were not recorded. A reported enquiry on these dimensions would have further aided the interpretation of the results. Third, control conditions could have been incorporated into the paradigm, such as depicting videos without any actors at all but relaying the same content. Addressing this limitation would have lent greater support to the discriminative validity of the study.

Nonetheless, this study has begun to unearth the neural mechanisms behind how *openness* modulates perceptions of interpersonal interaction (McCrae, 1996; McCrae and Sutin, 2009; Woo et al., 2014). By changing only the labels (i.e., relationship type) of the videos that participants were viewing, we found distinct activation patterns in the IFG, aPFC, and FEF as a function of one’s level of *openness*. As *openness* score increased, it was accompanied by elevated activation in these brain areas in response to videos in the *friendship* and *romantic* conditions, but decreased activation when viewing videos in the *siblings* condition. By distinguishing this pattern of response, we have identified the role of *openness* in modulating neurophysiological responses when perceiving social interactions belonging to different relationship categories. This places *openness* at the fore as an integral personality variable that not only dictates how we perceive social relationships, but possibly influences how we exhibit our affections and in turn interpret the affections we receive from different people in our lives. This fascinating finding sheds but a glimmer of understanding on how personality influences the ways in which people comprehend their social world, and how these perceptions take form on a neural level.

## Data Availability Statement

The datasets generated for this study can be found here: doi: 10.21979/N9/TSVWRR.

## Ethics Statement

The studies involving human participants were reviewed and approved by the Nanyang Technological University, Psychology Program, Ethics Committee. The patients/participants provided their written informed consent to participate in this study.

## Author Contributions

GE planned and supervised the entire study. AA, PR, PT, and MN collected the data and performed analyses. All authors wrote and commented on the manuscript.

## Conflict of Interest

The authors declare that the research was conducted in the absence of any commercial or financial relationships that could be construed as a potential conflict of interest.

## Footnotes

1 https://nirx.net/nirstar-1/
